# Anatomical Evaluation of the Alveolar Antral Artery in the Turkish Population: A Cone-Beam Computed Tomography Study

**DOI:** 10.7759/cureus.44163

**Published:** 2023-08-26

**Authors:** Rukiye Ciftci, Rabia Tasdemir, Ömer Faruk Cihan

**Affiliations:** 1 Department of Anatomy, Faculty of Medicine, Gaziantep Islam Science and Technology University, Gaziantep, TUR; 2 Department of Anatomy, School of Medicine, Gaziantep Islam Science and Technology University, Gaziantep, TUR; 3 Department of Anatomy, Gaziantep University, Gaziantep, TUR

**Keywords:** cone-beam computed tomography, edentulous, dentate, sex, diameter, alveolar antral artery

## Abstract

Background

Injury to the alveolar antral artery (AAA) is one of the most common complications in oral surgical procedures. This study aimed to determine the diameter and anatomical position of the AAA, establish reference values for the Turkish population, and contribute to the literature.

Methodology

The diameter of the AAA at the level of the first molar tooth, its distance from the sinus floor, its vertical distance to the alveolar crest, its oblique distance to the sinus floor, the width of the maxillary sinus, the thickness of the lateral sinus wall, the residual alveolar protrusion height, the residual alveolar protrusion width at the basal level, and the distance from the sinus lateral wall to the sinus floor were all measured using cone-beam computed tomography. Age, gender, and oral health were used to evaluate the collected data.

Results

The average age of the participants in the study was 42.63 ± 16.07 years. The average AAA diameter was 1.1 ± 0.25 mm, the average height of the residual alveolar protrusion was 0.44 ± 0.13 cm, the average width of the residual alveolar protrusion at the basal level was 0.79 ± 0.12 cm, and the average width of the residual alveolar ridge at the crest level was 0.55 ± 0.11 cm. No significant differences were observed in these parameters based on gender and dental status (p > 0.05). The average AAA distance to the sinus floor was 1.02 ± 0.26 cm, the average vertical distance to the alveolar crest was 1.21 ± 0.25 cm, the average oblique distance to the sinus floor was 1.38 ± 0.25 cm, the average maxillary sinus width was 1.63 ± 0.28 cm, the average thickness of the lateral sinus wall was 0.12 ± 0.06 cm, and the average distance from the sinus lateral wall to the sinus floor was 1.28 ± 0.22 cm. Significant differences based on gender were observed in all these parameters (p < 0.05). A significant difference was observed in the vertical distance from AAA to the alveolar crest and the oblique distance to the sinus floor based on dental status (p < 0.05), with shorter distances in dentate individuals. Only the AAA diameter showed a weak negative correlation with age (p < 0.05, 0.2 < r < 0.04).

Conclusions

The results obtained were within a reliable range for oral surgery. Detailed reference findings for the proximity and location of structures can be established for the Turkish population during dental surgery. It is recommended that physicians performing surgical interventions in the maxillary region carefully consider these reference values preoperatively.

## Introduction

The anatomical knowledge of the maxillary sinus and its surrounding tissues is of critical importance in various dental procedures, such as dental implants, sinus floor elevation, maxillary sinus grafting, Le Fort I fracture treatment, and Caldwell-Luc surgery [1-3]. Anastomosis between the posterior superior alveolar artery and the infraorbital artery can occur either intraosseously or extraosseously along the lateral wall of the maxillary sinus [4]. In the literature, some studies refer to both anastomotic branches as the alveolar antral artery (AAA), while others specifically label the intraosseous branch as AAA [1,4,5]. The AAA supplies blood to the lateral wall of the maxillary sinus and the posterior teeth, contributing not only to the nourishment of this area but also to graft integration and surgical site healing following procedures [1,4].

The vascular structure of the lateral sinus wall is influenced by factors such as age, gender, presence of teeth, and sinus volume [6].

When there is insufficient bone tissue for dental implants, a sinus lift procedure is performed [7]. To ensure a complication-free and successful sinus lift procedure, a thorough preoperative assessment of maxillary sinus anatomy is essential [7,8]. Cone-beam computed tomography (CBCT) is a preferred and reliable imaging method in maxillofacial imaging due to its superior image resolution [2,7-9].

In this study, intraosseous AAA diameter and distances to various anatomical points were measured on CBCT images to contribute to the establishment of reference values for dental practitioners during their preoperative assessments for surgery or implants. The assessment took into account age, gender, dental status (dentate or edentulous), symmetry, presence of polyps, and the open-closed state of the maxillary sinus. The goal was to conduct a detailed morphometric analysis to create references specific to the Turkish population.

## Materials and methods

Patient population

This retrospective study was conducted using CBCT images of individuals who had sought treatment for various reasons at the Faculty of Dentistry, Gaziantep University. Measurements were performed on images from a total of 405 patients, including 156 females and 249 males, aged between 18 and 90 years (mean age = 42.63 ± 16.07). Exclusion criteria encompassed missing, inconsistent, or confusing information about any planned measurement variables; images with artifacts that significantly hindered morphometric measurements; and CBCT images of cases with jaw diseases stemming from cysts and metabolic, developmental, or infectious causes that could affect the maxilla. Evaluated images were grouped in two different ways, namely, toothed and edentulous.

Morphometric measurements

**Figure 1:**
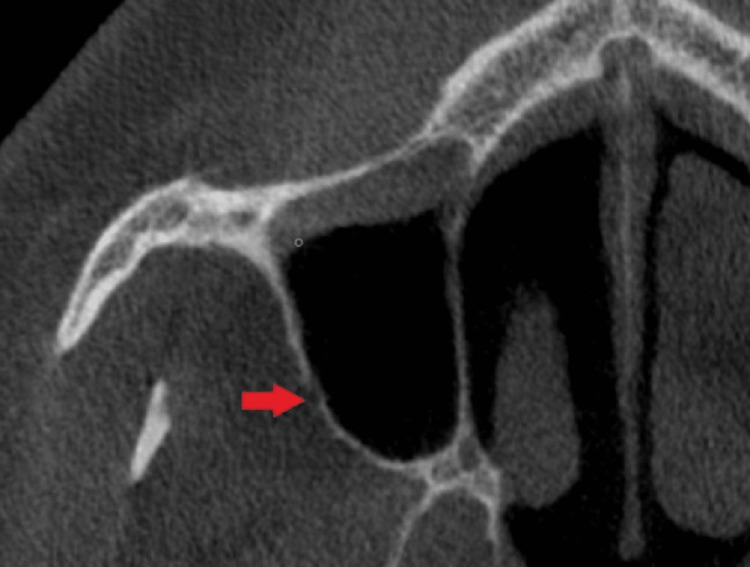
Diameter measurement of the AAA using CBCT. AAA = alveolar antral artery; CBCT = cone-beam computed tomography

**Figure 2:**
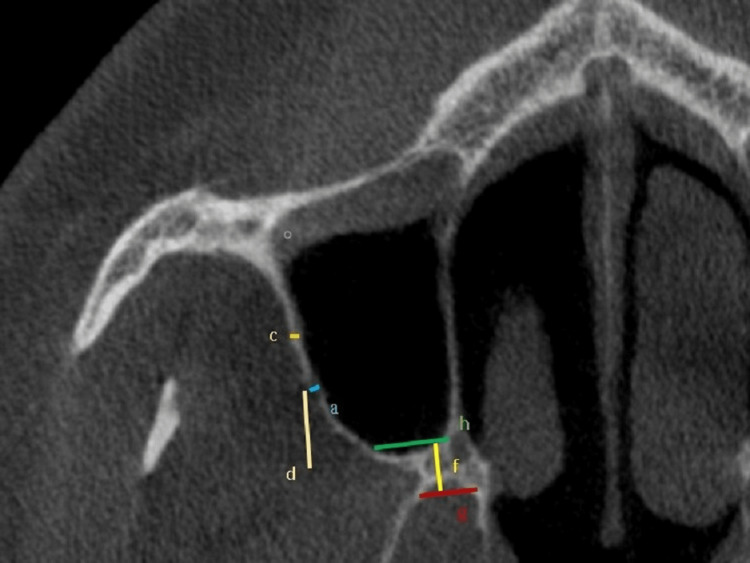
AAA diameter and other measurements made on CBCT. a = AAA diameter; c = thickness of the lateral sinus wall; d = distance from the AAA to the alveolar crest; f = height of the residual alveolar ridge; g = width of the residual alveolar ridge (basal level); h = width of the residual alveolar ridge (crest level). AAA = alveolar antral artery; CBCT = cone-beam computed tomography

**Figure 3:**
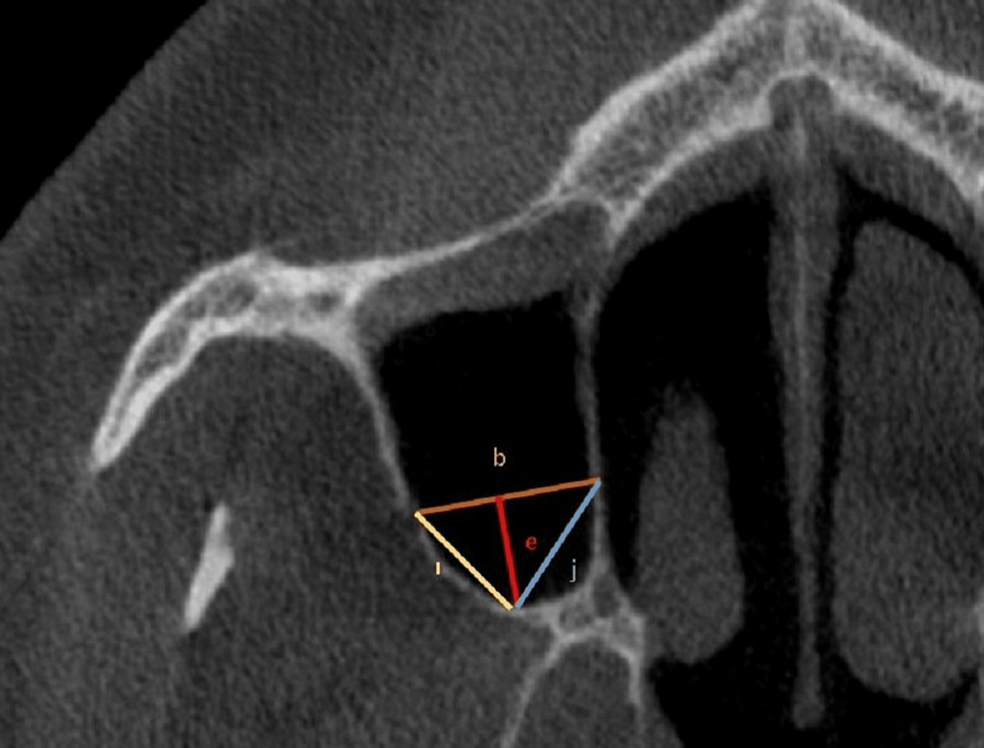
Other measurements made on the CBCT. b = maxillary sinus width; e = distance from the AAA to the sinus floor; i = oblique distance from the AAA to the sinus floor; j = distance from the sinus side wall to the floor. AAA = alveolar antral artery; CBCT = cone-beam computed tomography

The CBCT images were imported into the free program Horos v.4.0.0 (https://horosproject.org/) to enable the necessary measurements. Measurements on the coronal plane were used to discover the AAA for the first time (Figure 1). At the level of the first molar tooth, the diameter of the AAA was measured. Following these measurements, the maxillary sinus width, lateral sinus wall thickness, height and width of the basal residual alveolar protrusions, oblique distance from the AAA to the sinus floor, and width of the crest level residual alveolar ridge were measured (Figures 2, 3).

Statistical analysis

The SPSS software version 25 (IBM Corp., Armonk, NY, USA) was used to analyze the collected data. The Kolmogorov-Smirnov test was used to determine whether the data had a normal distribution. For comparison tests, a significance level (p) of 0.05 was applied. Non-parametric tests were used for further analysis because the variables’ absence of normal distribution (p > 0.05) precluded their use in the analysis. As the assumption of normality was not met, comparisons between independent binary groups were made using the Mann-Whitney U test. Spearman correlation was used to investigate correlations between numerical variables.

Compliance with ethical standards

This retrospective chart review study, including human subjects, was done in accordance with the ethical principles outlined in the 1964 Helsinki Declaration and its later amendments, as well as any comparable ethical standards. On May 30, 2023, the project received clearance from the Ethics Committee for Non-invasive Clinical Research at Gaziantep Islam Science and Technology University (approval number: 238.25.15).

## Results

Morphometric measurements

In total, 156 (38.5%) images used in the study were from females, whereas 249 (61.5%) were from males. Ages ranged from 18 to 90, with a mean of 42.63 ± 16.07 years (Table 1).

**Table 1:** Comparison of parameters by gender. Test = Mann-Whitney test; * = p < 0.05 indicating a statistically significant difference between the groups. AAA = alveolar antral artery

Parameters	Female	Male	Total	P-value
Age	42.73 ± 16.11	42.57 ± 16.07	42.63 ± 16.07	0.907
AAA diameter (mm)	1.09 ± 0.25	1.11 ± 0.25	1.1 ± 0.25	0.460
Distance from the AAA to the sinus floor (cm)	0.95 ± 0.23	1.07 ± 0.26	1.02 ± 0.26	0.001*
Vertical distance from the AAA to the alveolar crest (cm)	1.16 ± 0.26	1.25 ± 0.25	1.21 ± 0.25	0.001*
Maxillary sinus width (cm)	1.61 ± 0.3	1.65 ± 0.27	1.63 ± 0.28	0.036*
Thickness of the lateral sinus wall (cm)	0.11 ± 0.02	0.13 ± 0.08	0.12 ± 0.06	0.001*
Height of residual alveolar protrusion (cm)	0.43 ± 0.14	0.45 ± 0.13	0.44 ± 0.13	0.057
The width of the residual alveolar prominence at the basal level (cm)	0.79 ± 0.12	0.79 ± 0.12	0.79 ± 0.12	0.701
Oblique distance from the AAA to the sinus floor (cm)	1.32 ± 0.26	1.41 ± 0.24	1.38 ± 0.25	0.001*
Distance from the sinus side wall to the floor (cm)	1.24 ± 0.21	1.31 ± 0.22	1.28 ± 0.22	0.001*
Width of residual alveolar ridge at the crest level (cm)	0.54 ± 0.13	0.56 ± 0.1	0.55 ± 0.11	0.203

The mean diameter of the AAA was 1.09 mm in females and 1.11 mm in males. Based on gender or oral health, there was no discernible variation in diameter (p > 0.05). The distance from the AAA to the sinus floor was 0.95 ± 0.23 cm in females, 1.07 ± 0.26 cm in males, 1.00 ± 0.23 cm in dentate individuals, and 1.04 ± 0.27 cm in edentulous individuals. There was a significant gender difference (p < 0.05), but no statistical difference based on dental status (p > 0.05) (Figure 1, Table 1).

The vertical distance from the AAA to the alveolar crest was 1.16 ± 0.26 cm in females, 1.25 ± 0.25 cm in males, 1.18 ± 0.22 cm in dentate individuals, and 1.23 ± 0.27 cm in edentulous individuals. This parameter was significantly affected by gender and dental status (p = 0.05), with females and dentate individuals having lower scores (Figure 3, Table 1).

Males and females had a maxillary sinus width of 1.65 cm and 1.61 cm, respectively. Although there was no statistical difference depending on dental status (p > 0.05), there was a significant gender difference (p = 0.05) (Figure 3, Table 1).

The thickness of the lateral sinus wall was 0.11 ± 0.02 cm in females and 0.13 ± 0.08 cm in males. There was a significant gender difference favoring males (p < 0.05), but no significant difference based on dental status (p > 0.05) (Figure 2, Table 1).

The height of the residual alveolar protrusion was 0.44 ± 0.13 cm, and the width at the basal level was 0.79 ± 0.12 cm. There were no significant differences based on gender or dental status (p > 0.05) for both measurements (Figure 2, Table 1).

The oblique distance from the AAA to the sinus floor was 1.32 ± 0.26 cm in females, 1.41 ± 0.24 cm in males, 1.35 ± 0.24 cm in dentate individuals, and 1.39 ± 0.26 cm in edentulous individuals. Significant variations were seen between gender and dental status (p = 0.05), with males and edentulous people having higher values (Figure 3, Table 1).

The measurement between the sinus lateral wall and the sinus floor was 1.28 ± 0.22 cm, with females measuring 1.24 ± 0.21 cm and males measuring 1.31 ± 0.22 cm. However, there was no significant difference based on dental status (p > 0.05), with only a significant gender difference favoring men (p < 0.05) (Figure 3, (Table 1).

The width of the residual alveolar ridge at the crest level was 0.55 ± 0.11 cm, and no significant differences were observed among the groups (p > 0.05) (Figure 2, Table 2).

**Table 2:** Comparison of parameters according to gear-toothless conditions. Test = Mann-Whitney test; * = p < 0.05 indicating a statistically significant difference between the groups. AAA = alveolar antral artery

Parameters	Dentate	Edentulous	Total	P-value
Age	29.52 ± 9.67	50.26 ± 14	42.63 ± 16.07	0.000
AAA diameter (mm)	1.11 ± 0.22	1.09 ± 0.26	1.1 ± 0.25	0.204
Distance from the AAA to the sinus floor (cm)	1 ± 0.23	1.04 ± 0.27	1.02 ± 0.26	0.075
Vertical distance from the AAA to the alveolar crest (cm)	1.18 ± 0.22	1.23 ± 0.27	1.21 ± 0.25	0.033*
Maxillary sinus width (cm)	1.64 ± 0.28	1.63 ± 0.28	1.63 ± 0.28	0.442
Thickness of the lateral sinus wall (cm)	0.12 ± 0.04	0.12 ± 0.08	0.12 ± 0.06	0.789
Height of residual alveolar protrusion (cm)	0.44 ± 0.14	0.45 ± 0.13	0.44 ± 0.13	0.443
The width of the residual alveolar prominence at the basal level (cm)	0.8 ± 0.1	0.78 ± 0.13	0.79 ± 0.12	0.105
Oblique distance from the AAA to the sinus floor (cm)	1.35 ± 0.24	1.39 ± 0.26	1.38 ± 0.25	0.023*
Distance from the sinus side wall to the floor (cm)	1.27 ± 0.21	1.29 ± 0.22	1.28 ± 0.22	0.188
Width of residual alveolar ridge at the crest level (cm)	0.57 ± 0.12	0.55 ± 0.11	0.55 ± 0.11	0.147

There was a weak negative relationship between AAA diameter and age (p < 0.05; 0.20) when assessing the correlation between diameter and distance measurements with age (Figure 1, Table 2).

The distance from the sinus lateral wall to the sinus floor, the height of the residual alveolar protrusion, the width of the residual alveolar protrusion at the basal level, the thickness of the lateral sinus wall, and the vertical distance between the AAA and the alveolar crest showed weak positive correlations with the AAA diameter (p < 0.05; 0.20) (Figure 3, Table 3).

**Table 3:** Correlation of parameters with age and each other. r = Spearman rank correlation coefficient; * = p < 0.05 indicating a statistically significant difference between the groups. AAA = alveolar antral artery

Points	Value	Age	AAA diameter (mm)	Distance from the AAA to the sinus floor (cm)	Vertical distance from the AAA to the alveolar crest (cm)	Maxillary sinus width (cm)	Thickness of the lateral sinus wall (cm)	Height of residual alveolar protrusion (cm)	The width of the residual alveolar prominence at the basal level (cm)	Oblique distance from the AAA to the sinus floor (cm)	Distance from the sinus side wall to the floor (cm)	Width of residual alveolar ridge at the crest level (cm)
AAA diameter (mm)	r	-0.206*	1.000	0.252**	0.218*	0.047	0.296**	0.200**	0.256**	0.096	0.206*	0.070
	p	0.034	0.000	0.002	0.017	0.349	0.000	0.000	0.000	0.053	0.032	0.257
Distance from the AAA to the sinus floor (cm)	r	0.066	0.252**	1.000	0.767**	0.519**	0.231**	0.262**	0.266**	0.848**	0.873**	-0.003
	p	0.284	0.002	0.000	0.000	0.000	0.000	0.001	0.001	0.000	0.000	0.954
Vertical distance from the AAA to the alveolar crest (cm)	r	0.094	0.218*	0.767**	1.000	0.406**	0.281**	0.231**	0.232**	0.698**	0.683**	-0.038
	p	0.059	0.017	0.000	0.000	0.000	0.000	0.000	0.008	0.000	0.000	0.451
Maxillary sinus width (cm)	r	-0.079	0.047	0.519**	0.406**	1.000	-0.042	0.232**	0.236**	0.712**	0.658**	-0.003
	p	0.214	0.349	0.000	0.000	0.000	0.395	0.008	0.000	0.000	0.000	0.954
Thickness of the lateral sinus wall (cm)	r	-0.042	0.296**	0.231**	0.281**	-0.042	1.000	-0.003	0.256**	0.213*	0.221*	0.203**
	p	0.403	0.000	0.000	0.000	0.395	0.000	0.958	0.002	0.023	0.015	0.000
Height of residual alveolar protrusion (cm)	r	0.044	0.200**	0.262**	0.231**	0.232**	-0.003	1.000	0.071	0.242**	0.242**	0.060
	p	0.375	0.000	0.001	0.000	0.008	0.958	0.000	0.254	0.000	0.004	0.229
The width of the residual alveolar prominence at the basal level (cm)	r	-0.094	0.256**	0.266**	0.232**	0.236**	0.256**	0.071	1.000	0.290**	0.258**	0.435**
	p	0.058	0.000	0.001	0.008	0.000	0.002	0.254	0.000	0.000	0.000	0.000
Oblique distance from the AAA to the sinus floor (cm)	r	0.080	0.096	0.848**	0.698**	0.712**	0.213*	0.242**	0.290**	1.000	0.840**	-0.010
	p	0.207	0.053	0.000	0.000	0.000	0.023	0.000	0.000	0.000	0.000	0.838
Distance from the sinus side wall to the floor (cm)	r	0.019	0.206*	0.873**	0.683**	0.658**	0.221*	0.242**	0.258**	0.840**	1.000	-0.015
	p	0.704	0.032	0.000	0.000	0.000	0.015	0.004	0.000	0.000	0.000	0.759
Width of residual alveolar ridge at the crest level (cm)	r	-0.067	0.070	-0.003	-0.038	-0.003	0.203**	0.060	0.435**	-0.010	-0.015	1.000
	p	0.276	0.257	0.954	0.451	0.954	0.000	0.229	0.000	0.838	0.759	0.000

The vertical distance from the AAA to the alveolar crest and the horizontal distance from the AAA to the sinus floor were both moderately positively associated with maxillary sinus width (p = 0.05; 0.40 < r < 0.60). The correlation analysis for the variables is summarized in Table 3 (Figure 2, Table 3).

## Discussion

In sinus floor elevation procedures, the risk of bleeding associated with the diameter of the AAA is 57% when the diameter is within the range of 1-2 mm; this risk increases as the diameter increases [10]. Varela-Centelles et al. [10] reported that AAA diameter ranging between 1 and 2 mm was associated with gender, maxillary sinus width, and thickness of the maxillary sinus lateral wall. They reported that as maxillary sinus width and lateral wall thickness increase, the diameter of the vessel also increases and that males tend to have larger diameters. In a prevalence study conducted among the Thai population, Laovoravit et al. [11] reported an average AAA diameter of 16.02 ± 3.94 mm at the level of the first molar. A study that examined AAA anatomy in edentulous patients reported an average diameter of 1.2-1.5 mm and a distance from the AAA to the sinus floor ranging from 7.9 to 12 mm [8]. In a study comparing the morphology of AAA in dentate and edentulous individuals, the diameter at the level of the first molar was 1.32 ± 0.34 mm in the dentate group and 1.00 ± 0.20 mm in the edentulous group. Both groups exhibited a negative correlation between age and AAA diameter [12]. In this study, the AAA diameter was found to be an average of 1.1 ± 0.25 mm, and no differences were observed based on gender or dental status. The values obtained in this study are consistent with the literature and fall within a reliable range for surgical interventions.

During osteotomy surgeries, anatomical parameters such as AAA diameter, position, distance from the alveolar crest, and distance from the sinus floor may cause problems [6]. Varela-Centelles et al. [6] reported that the average distances between AAA and the sinus floor were 7.66 mm, 15.26 mm, 7.76 mm, 10.18 mm, and 6.68 mm, respectively. The distance between AAA and the alveolar crest was also reported to be 7.66 mm on average. These measurements, according to them, were within acceptable surgical bounds. The findings of our investigation were consistent with previous studies.

Zhou et al. [13] reported that the distance from AAA to the sinus floor was 8.00 ± 2.68 mm, with a significant difference between dentate and edentulous individuals. They also measured the lateral sinus wall thickness as 2.03 ± 0.91 mm and found it to be significantly larger in males compared to females. In our study, this distance was larger in males than females, which was higher than that reported in the literature.

According to a study examining AAA position in the South Indian population, the distance between AAA and the alveolar crest at the level of the first molar was 16.08 ± 0.16 mm in edentulous individuals and 17.36 ± 0.51 mm in dentate patients. They discovered that 80% of the individuals who were studied had a consistent gap between the AAA and the alveolar crest [14]. Another study by Varela-Centelles et al. [15] on preoperative patients revealed that the lateral sinus wall thickness averaged 2.31 mm, the residual alveolar protrusion measured 7.44 mm in height, 10.06 mm in width at the basal level, and 6.62 mm at the crest level. The maxillary sinus breadth was also reported to be 12.60 mm. They observed that raising these factors increased the possibility of finding the AAA [15]. For the lateral sinus wall thickness and the breadth of the residual alveolar protrusion at basal and crest levels, we observed that our findings were quite close to those described in the literature, although we discovered that the maxillary sinus width was less than that described in the literature. This mismatch is due to the gender-based data classification used in this study. In a study investigating the anatomical structure of the maxillary sinus on cadavers, the prevalence of AAA diameter less than 1 mm was reported as 55.3%, between 1 and2 mm was 40.4%, and greater than 2 mm was 4.3%. They indicated that a total of 94.7% had a surgically reliable artery diameter [16]. In the same study, the distance from the AAA to the alveolar crest at the level of the first molar was reported as 11.25 ± 2.99 mm, and the height of the residual alveolar protrusion was reported as 3.60 ± 1.28 mm [16]. In our study, the AAA diameter below 1 mm was 37.5%, and between 1 and 2 mm was 62.5%. We found that AAA diameter between 1 and 2 mm was more frequent in our study. We attribute this result to the larger sample size compared to the literature.

In a study examining AAA on CT images, they found that the canal diameter was less than 1 mm in 26% of cases, between 1 and 2 mm in 22.1%, and between 2 and 3 mm in 6.7%. They reported a positive correlation between diameter and age. The distance from the AAA to the alveolar crest at the level of the first molar was measured as 16.92 ± 4.46 mm [17]. In another study that examined AAA anatomy in dentate and partially edentulous patients grouped by age, the left AAA diameter waas an average of 1.19 ± 0.40 mm and the right AAA diameter was an average of 1.30 ± 0.42 mm. The distance from the AAA to the alveolar crest at the level of the first molar was measured as an average of 11.65 ± 2.37 mm on the left and 11.60 ± 2.66 mm on the right [18]. A study aiming to determine the anatomical reference position of AAA preoperatively reported the distance from the AAA to the alveolar crest to be an average of 19.2 ± 3.5 mm, and the distance to the sinus floor to be an average of 8.6 ± 3.3 mm [19]. In a study that examined the anatomical structure of the AAA according to gender, dental status, and ethnic differences, the average AAA diameter was reported as 0.91 ± 0.56 mm, and the distance from AAA to the sinus floor at the level of the first molar was reported as 7.03 ± 3.37 mm. They stated that both parameters were not affected by gender, dental status, or ethnicity [20]. In a study investigating the position of the AAA in the Korean population, the distance from the AAA to the alveolar crest was an average of 16.64 ± 2.58 mm, and they reported that this distance was statistically significant in the dentate group. In the same study, the distance from the AAA to the sinus floor was reported as an average of 7.71 ± 2.17 mm, and they did not find a significant difference based on dental status [21,22].

In this study, among the reference distances determined for the anatomical position of AAA, the distance from the AAA to the sinus floor, maxillary sinus width, lateral sinus wall thickness, and the distance from the sinus lateral wall to the sinus floor were significantly higher in males compared to females. On the other hand, the vertical distance from the AAA to the alveolar crest and the oblique distance from the AAA to the sinus floor showed significant differences based on gender and dental status. Overall, the findings of this study are in line with the literature, and it can be concluded that the position of the AAA falls within a surgically reliable reference range.

Limitations

For the morphometric measurements, patients in whom the AAA could be visualized were selected. Therefore, a comparison could not be made with individuals in whom the AAA was not visible. Another limitation is that measurements were taken primarily at the level of the first molar, where the AAA is most clearly visible and osteotomy procedures are frequently performed. As a result, comparisons with measurements taken at other tooth locations, as reported in the literature, could not be performed. A larger and multicenter study involving a broader population could provide more reliable results for the Turkish population.

## Conclusions

In this study, only the height of the residual alveolar crest and its width at both the basal and crest levels were not affected by gender and dental status. The reference point for the AAA can be determined based on these levels in preoperative evaluations. As other parameters showed significant variations based on gender and dental status, dentists should consider these factors in their assessments.

No other study focusing on a detailed analysis of the anatomical position of the AAA in the Turkish population has been identified previously. In this study, the morphometric characteristics of the maxillary sinus and AAA were comprehensively investigated by establishing 10 reference distances. Additionally, the morphological features of the maxillary sinus were examined to determine whether they influenced the morphometry. The findings are believed to contribute to the literature on the Turkish population.

## References

[1] Yusof MY, Mah MC, Reduwan NH, Kretapirom K, Affendi NH (2020). Quantitative and qualitative assessments of intraosseous neurovascular canals in dentate and posteriorly edentulous individuals in lateral maxillary sinus wall. Saudi Dent J.

[2] Shahidi S, Zamiri B, Momeni Danaei S, Salehi S, Hamedani S (2016). Evaluation of anatomic variations in maxillary sinus with the aid of cone beam computed tomography (CBCT) in a population in south of Iran. J Dent (Shiraz).

[3] Chitsazi MT, Shirmohammadi A, Faramarzi M, Esmaieli F, Chitsazi S (2017). Evaluation of the position of the posterior superior alveolar artery in relation to the maxillary sinus using the cone-beam computed tomography scans. J Clin Exp Dent.

[4] Pimkhaokham A, Aung CM, Panmekiat S (2016). The study of the alveolar antral artery canal in using cone beam computed tomography. M Dent J.

[5] Alves N, Torres C, Deana NF, Garay I (2020). Analysis of the presence, location and morphometry of the alveolar antral artery by cone-beam computed tomography in Chilean adults. Int J Morphol.

[6] Varela-Centelles P, Loira-Gago M, Gonzalez-Mosquera A, Seoane-Romero JM, Garcia-Martin JM, Seoane J (2016). Distance of the alveolar antral artery from the alveolar crest. Related factors and surgical considerations in sinus floor elevation. Med Oral Patol Oral Cir Bucal.

[7] Rahpeyma A, Khajehahmadi S (2015). Open sinus lift surgery and the importance of preoperative cone-beam computed tomography scan: a review. J Int Oral Health.

[8] Albuquerque DP, Manhães Junior LR, Silva MB, Francischone CE, Franco A, Junqueira JL (2021). Alveolar antral artery in edentulous patients and their visualization through cone beam computed tomography. Morphologie.

[9] Yalçın M, Demirkol M (2023). Clinical significance of CBCT findings in the treatment of maxillary cysts expanded into the nasal and sinus cavities. Eur J Ther.

[10] Varela-Centelles P, Seoane J, Loira-Gago M, González-Mosquera A, Seoane-Romero JM (2017). Diameter of alveolar antral artery in the lateral sinus wall: study of related factors. Br J Oral Maxillofac Surg.

[11] Laovoravit V, Kretapirom K, Pornprasertsuk-Damrongsri S (2021). Prevalence and morphometric analysis of the alveolar antral artery in a group of Thai population by cone beam computed tomography. Oral Radiol.

[12] Kolte RA, Kolte AP, Rahate PS, Bawankar PV (2021). Association of location and diameter of alveolar antral artery to crest of alveolar bone in dentate and partially edentulous patients - a cone-beam computed tomography study. J Indian Soc Periodontol.

[13] Zhou Q, Qiao F, Zhu D (2023). The radiological evaluation of the anatomy of the alveolar antral artery and the lateral wall thickness using cone-beam computed tomography: a retrospective study [in press]. Curr Med Imaging.

[14] Soundarajan S, Kaarthikeyan G (2023). Evaluation of alveolar antral anastomosis in south Indian population using cone beam computed tomography: a prospective study. Oral Radiol.

[15] Varela-Centelles P, Loira M, González-Mosquera A, Romero-Mendez A, Seoane J, García-Pola MJ, Seoane-Romero JM (2020). Study of factors influencing preoperative detection of alveolar antral artery by CBCT in sinus floor elevation. Sci Rep.

[16] Rosano G, Taschieri S, Gaudy JF, Weinstein T, Del Fabbro M (2011). Maxillary sinus vascular anatomy and its relation to sinus lift surgery. Clin Oral Implants Res.

[17] Mardinger O, Abba M, Hirshberg A, Schwartz-Arad D (2007). Prevalence, diameter and course of the maxillary intraosseous vascular canal with relation to sinus augmentation procedure: a radiographic study. Int J Oral Maxillofac Surg.

[18] Rathod R, Singh MP, Nahar P, Mathur H, Daga D (2022). Assessment of pathway and location of posterior superior alveolar artery: a cone-beam computed tomography study. Cureus.

[19] Takahashi A, Kamada K, Kudoh T (2022). Evaluation of anatomical references for locating the course of the posterior superior alveolar artery for dental implant surgery. Int J Oral Maxillofac Surg.

[20] Tran TB, Estrin NE, Saleh MH, Yoon TY, Tattan M, Wang HL (2021). Evaluation of length and location of the maxillary sinus intraosseous artery using computerized tomography. J Periodontol.

[21] Park WH, Choi SY, Kim CS (2012). Study on the position of the posterior superior alveolar artery in relation to the performance of the maxillary sinus bone graft procedure in a Korean population. J Korean Assoc Oral Maxillofac Surg.

[22] Senol D (2023). Analysis of the effects of total pneumatized turbinate volume on septum deviation, maxillary sinus volume, and maxillopalatal parameters: a multidetector computerized tomography study. J Anat Soc India.

